# State of the field: An informatics-based systematic review of the SOD1-G93A amyotrophic lateral sclerosis transgenic mouse model

**DOI:** 10.3109/21678421.2015.1047455

**Published:** 2015-05-22

**Authors:** Renaid B. Kim, Cameron W. Irvin, Keval R. Tilva, Cassie S. Mitchell

**Affiliations:** ^a^ Department of Biomedical Engineering, Georgia Institute of Technology & Emory University, Atlanta, Georgia, USA

**Keywords:** Mitochondria, excitotoxicity, gliosis, protein aggregation, reactive oxygen species, rotarod, calcium, neuropathology

## Abstract

Numerous sub-cellular through system-level disturbances have been identified in over 1300 articles examining the superoxide dismutase-1 guanine 93 to alanine (SOD1-G93A) transgenic mouse amyotrophic lateral sclerosis (ALS) pathophysiology. Manual assessment of such a broad literature base is daunting. We performed a comprehensive informatics-based systematic review or ‘field analysis’ to agnostically compute and map the current state of the field. Text mining of recaptured articles was used to quantify published data topic breadth and frequency. We constructed a nine-category pathophysiological function-based ontology to systematically organize and quantify the field's primary data. Results demonstrated that the distribution of primary research belonging to each category is: systemic measures an motor function, 59%; inflammation, 46%; cellular energetics, 37%; proteomics, 31%; neural excitability, 22%; apoptosis, 20%; oxidative stress, 18%; aberrant cellular chemistry, 14%; axonal transport, 10%. We constructed a SOD1-G93A field map that visually illustrates and categorizes the 85% most frequently assessed sub-topics. Finally, we present the literature-cited significance of frequently published terms and uncover thinly investigated areas. In conclusion, most articles individually examine at least two categories, which is indicative of the numerous underlying pathophysiological interrelationships. An essential future path is examination of cross-category pathophysiological interrelationships and their co-correspondence to homeostatic regulation and disease progression.

## Introduction

Amyotrophic lateral sclerosis (ALS) is characterized by progressive neurodegeneration of the motor neurons, which leads to muscle paralysis, respiratory deficiency, and eventually death. Mutations of the superoxide (copper-zinc) dismutase-1 (SOD1) gene have been identified as contributors to familial ALS, which accounts for approximately 5–10% of all ALS cases (1). The SOD1-G93A (glycine 93 to alanine) mutation is a comparatively rare ALS mutation in humans, but it is the most studied and published mutation within experimental transgenic ALS mouse models (2,3). The SOD1-G93A transgenic ALS model's popularity is largely due to its ALS symptom reproducibility and its widespread availability for purchase from The Jackson Laboratory (jaxmice.jax.org). At the end of the 2014 year, searching for ‘Amyotrophic Lateral Sclerosis’ AND ‘G93A’ in PubMed returned approximately 1300 articles, and the tally was actually greater since not every article using the SOD1-G93A model specifically cites ‘G93A’ in the PubMed-searchable locations (i.e. title, abstract, etc.).

The SOD1-G93A mouse model has been utilized to identify numerous deficits and impairments contributing to or the direct result of the mutation's associated ALS pathophysiology. Briefly, such deficits comprise the following: apoptosis, including changes in pro- and anti-apoptotic signals (4); axonal transport of mitochondria and other key cargoes (5); aberrant cellular chemistry such as reduced enzyme activity and metal mishandling (6); energetics, including disturbances of the physical and functional properties of mitochondria, ATP production and calcium homeostasis (7); genetic damage, including changes in mRNA or DNA; inflammation, including the migration of reactive astrocytes and microglia (8); oxidative stress, resulting from the build-up of free radicals (9); proteomics, characterized by the accumulation of misfolded SOD1 aggregates (10); systemic impairments, including overall system-level neuromuscular function and non-neuromuscular contributors (3). The phenotype severity and disease progression of the SOD1-G93A transgenic mouse is largely dependent upon the transgene copy number (typically denoted as ‘high’ versus ‘low’). The overwhelming majority of SOD1-G93A transgenic mouse studies have used a high copy model, which has an average onset range of 85–100 days and endpoint of 120–160 days (3).

Knowing the distribution and categorization of primary data is a key step towards both consolidating current knowledge and planning new research (11). However, with so many articles covering such an expansive and complex pathophysiology, it is difficult to manually determine what has and has not been examined in the SOD1-G93A transgenic ALS mouse field. Moreover, while traditional literature reviews help in digesting the details of published data, ideas, or mechanisms, their content does not necessarily quantitatively align with what actual primary data exist for a given topic or theorem. Authors of traditional literature reviews must subjectively determine what topics are reported based on the author's exposure to the field. Automated informatics-based systematic reviews or ‘field analyses’ overcome the traditional limitations of manual literature reviews by comprehensively and agnostically searching the primary data of every available article to quantify the breadth and depth of researched topics. The result is an objective map of the overall literature that structurally organizes and numerically identifies topical areas of prevalent data as well as disparate or thinly investigated areas where primary data are sparse.

Consequently, the goals of this informatics-based systematic review of the SOD1-G93A mouse model were to: 1) determine the published breadth and frequency of research topics; 2) systematically organize and categorize primary data articles using a pathophysiological function ontology; and 3) consolidate current knowledge and highlight corresponding future research paths.

## Materials and methods

The general method included finding SOD1-G93A articles; recapturing data from the article entities; devising and testing a term-category dictionary for identifying research terms/topics and for ontological categorization; searching the article entities to determine the frequency of primary data terms/research topics; assessment of the publication frequency and distribution within the pathophysiological ontological categories.

### Inclusion and exclusion criteria

To obtain the initial primary article selection pool, PubMed searches were conducted in October 2014 to find all published articles with (‘Amyotrophic Lateral Sclerosis’ or ‘ALS’) in the title or abstract and (‘transgenic mouse’ or ‘G93A’) in the title or abstract. Initial primary article selection pool exclusion criteria consisted of: non-English language articles; articles for which full-text pdf downloads were unavailable; and articles labeled as literature reviews. Articles were either downloaded using PubMed Central or from e-journal subscriptions available from the libraries of Georgia Institute of Technology and Emory University. Using these methods, less than 3% of the eligible initial article pool was unavailable for download. Based on these initial criteria, 1997 articles were eligible for inclusion in the initial primary article selection pool.

To obtain the final article pool utilized to conduct this study, within-article keyword searches were performed to find articles that contained ‘G93A’ in at least one of the following locations: article title, abstract, figure caption, or within the figure text (see Data recapture for details). The final article pool consisted of 1339 articles, all of which were included in the field analysis.

It should be noted that the overwhelming majority of studies do not distinguish between strain and transgene copy number in the searchable article entities (and many articles do not mention them at all, even in the full-text methods). Thus, articles were not included or excluded based on transgene copy number (e.g. high, low) or strain (e.g. B6SJL, C57BL/6), i.e. our article pool represents a combined assessment of research topics in the overall SOD1-G93A transgenic mouse field.

### Data recapture

Data were recaptured from the following article locations, referred to as entities: article title, abstract, figure captions, and within figure text. ‘Within figure’ text included any text labeled on or within a figure or table, e.g. the x-y axis labels, bar graph categorical labels, legends, etc. Recaptured data were obtained from downloaded full-text pdf files. Abstract, title, and reference information was exported directly from PubMed. Figure captions and within figure text was manually scraped from the full-text pdf articles using a standard keyboard copy and paste command (12). Any special characters that did copy correctly were manually revised. A quality control team independently assessed all data recapture to ensure complete accuracy. Recaptured data were transcribed into a custom project-specific searchable relational database (www.pathology-dynamics.org). The database is implemented in Filemaker 13 Pro Advanced (Filemaker, Inc.).

### Term-category dictionary

A dictionary of corresponding terms and categories (referred to as a term-category dictionary) was constructed that assigned frequent SOD1-G93A pathophysiology article terms and phrases to their most probable ontological category. The term- category dictionary allowed for automated searching of recaptured text and labeling of the articles entity's and the overall article's most probable ontological categories.

The chosen ontological categories were based on a previously published scheme (2) developed from a meta-analysis of SOD1-G93A traditional literature review articles. The ontology was used to categorize primary research data based on underlying pathophysiological function. The ontological categories consisted of: Apoptosis, Axonal Transport, Chemistry, Energetics, Excitability, Genetic Damage, Inflammation, Oxidative Stress, Proteomics and Systemic. The ontological categories are defined in detail in the Results and Discussion section.

To determine the most frequent keywords and phrases in the SOD1-G93A articles, word and phrase frequency analysis was performed using freely available software from WriteWords. Approximately 6500 different terms and phrases were identified and sorted by their number of appearances in each recaptured entity and by their total number of appearances in the articles. Non-scientific words insignificant to the analysis (e.g. of, in, and, etc.) were immediately excluded. Subsequently, a group of trained researchers in SOD1-G93A pathophysiology preliminarily labeled the most frequent 2000 terms by their most likely ontological category.

After performing the first ontological test set search, some individual terms were combined to provide for better specificity (see details in Ontological Test sets). Ultimately, 670 terms and phrases were selected for inclusion of the term-category dictionary (Supplementary Table I to be found online at http://informahealthcare.com/doi/abs/10.3109/21678421.2015.1047455).

### Field searches

Each keyword or phrase in the term-category dictionary was searched in the recaptured data article entities (article title, abstract, figure caption, and within figure text). If the search keyword was a single word, a whole word search was performed. For a phrase, a whole word search was performed for each word but not necessarily in the order of the words, e.g. ‘copper concentration’ and ‘concentration of copper’ were detected upon searching for ‘copper concentration’. If the search was positive, the figure and article was labeled by the corresponding ontological category of that term. The categories identified in the figure caption and within figure text were combined to represent each individual figure's ontological categorization. The categories identified in the article title, figure caption, and within figure text were combined to represent the overall categorization of each article. It should be noted that the abstract was ultimately excluded from the overall article categorization due to the number of false-positive hits it produced (see Test sets). Similarly, the ontological category, Genetic Damage, was individually excluded from the final field analysis results to decrease false-positives; corresponding articles were re-categorized according to the cited location of genetic damage (see Test sets).

### Test sets

Test sets of SOD1-G93A figures and corresponding articles were constructed to determine which recaptured article entities should be searched and to evaluate the term-category dictionary. Each test set minimally consisted of 500–600 figures from 100 different SOD1-G93A articles, which included primary data representing each ontological category. For the purpose of evaluating the term-category dictionary, the test set's ontological categorization was separately and manually determined by independent visual inspection of the article's primary data by five trained SOD1-G93A pathophysiology researchers.

Evaluation included measures of sensitivity and specificity. Sensitivity (the ability of a test to identify a condition correctly) and specificity (the ability of a test to exclude a condition correctly) are often used to assess the capability of a search to produce accurate results. Sensitivity is defined as: number of true-positives (TP) divided by the sum of the number of true-positives and false-negatives (FN): TP/ [TP + FN]. Specificity is defined as the number of true- negatives divided by the sum of true-negatives and false-positives: TN/[TN + FP]. While having both a high sensitivity and specificity is ideal, realistically optimization is typically favored towards one or the other depending on the search/test outcome goal, i.e. whether it is more important that the search/test includes or excludes a condition. Given that our protocol searches multiple terms per entity and thus allows multiple categories to be assigned, specificity (the ability to exclude) was given greater priority in the test set design and assessment.

We assessed the article entities’ ability to correctly represent the primary data contained within the article. Searching the abstract text resulted in > 50% false-positives, i.e. over 50% of the abstracts contained key terms or phrases that were either not represented/relevant to the article's primary data or were not present in the article's figure caption or within figure text. If the abstracts were to be used as part of the determination of the articles’ overall categorization, their false-positive terms would result in the addition of non-relevant categories. In contrast, searching the figure captions and within figure text resulted in < 2% false-positives and article titles < 4%. Therefore, as noted in field searches, the abstract search was excluded from the final ontological categorization of an article.

Subsequently, we assessed the ability of each ontological category to represent the corresponding article's primary data. All categories had greater than 95% specificity with the exception of Genetic Damage. The category Genetic Damage consisted of many general terms (see Supplementary Table I to be found online at http://informahealthcare.com/doi/abs/10.3109/21678421.2015.1047455), which resulted in low specificity (< 70%). Fortunately, genetic damage is typically measured in a specific location, organelle or pathway. Thus, articles containing primary data that examined genetic damage were labeled with the category(ies) that corresponded to the location or physiology affected by the genetic damage (e.g. mitochondrial mRNA damage → Energetics).

Finally, an ontological test set was also utilized to assess the term-category dictionary itself. An initial test to identify false-positive and negatives resulted in 86.1% sensitivity and 79.0% specificity for articles and 81.5% sensitivity and 87.6% specificity for figures. Care was taken to increase specificity by combining terms that created numerous false-positives into more specific phrases (e.g. aggregation → protein aggregation). After correction, another test set was utilized to evaluate the final dictionary's accuracy: 87.9% sensitivity and 99.9% specificity for papers and 77.9% sensitivity and 97.5% specificity for figures. Given that specificity was the priority, an overall specificity > 98% was considered acceptable for the study goals.

## Results and discussion

We first present the field analysis results, including the overall distribution of research articles with primary data belonging to each of the nine ontological categories: Apoptosis, Axonal Transport, Chemistry, Energetics, Excitability, Inflammation, Oxidative Stress, Proteomics and Systemic. Subsequently, the quantitative topical distribution of research articles in each of the individual categories is presented and explained. Finally, we conclude with a discussion on categorical relationships and future directions.

### Overall SOD1-G93A Field Analysis

**Figure 1.**
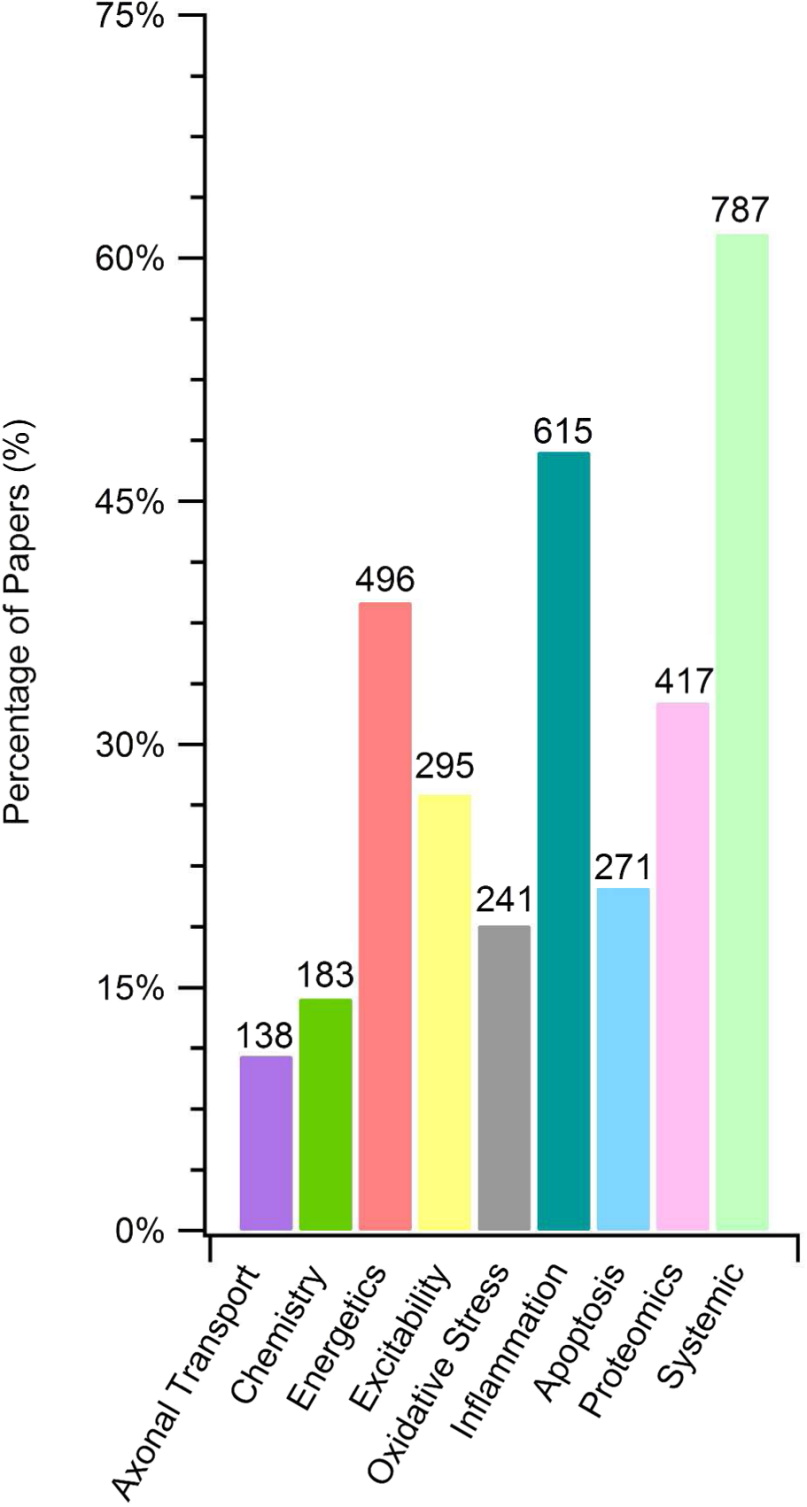
SOD1-G93A transgenic mouse model research categorized by article topic frequency based on a nine-category pathophysiological function ontology. For each category, the graph illustrates the absolute article count and percentage of the total SOD1-G93A articles with primary data based on word frequency searches of the title, figure captions, and within figure text. Articles are typically classified under two or three categories due to the many inherent biological and pathological inter-category relationships.

We performed a field analysis based on key word searches of article titles, figure captions, and within figure text to examine the prevalence of the different types of pathophysiological research in the SOD1-G93A ALS transgenic mouse model. The order of overall prevalence of primary data corresponding to the defined ontological categories of the SOD1-G93A ALS published literature is as follows: 1) systemic and functional measures, 59%; 2) inflammation, 46%; 3) cellular energetics, 37%; 4) proteomics, 31%; 5) excitability, 22%; 6) apoptosis, 20%; 7) oxidative stress,18%; 8) aberrant cellular chemistry, 14%; and 9) axonal transport, 10%. Figure 1 shows the distribution of articles across the nine categories as a percentage of the total SOD1-G93A articles along with their absolute article count.

All included primary articles and their figures were labeled with at least one category using the term-category dictionary. However, given the complexity of the SOD1-G93A pathology, most articles actually belonged to more than one category. For example, a primarily proteomic study examining protein aggregation in conjunction with iron sulfur protein (ISP) belongs to both chemistry (due to ‘iron’) and proteomics (due to ‘protein aggregation’). The average number of categories labeled for each article using the term-category dictionary search was 2.6 with a standard deviation of ± 1.6. Multi-category assignment to SOD1-G93A articles is, in large part, due to the numerous cross-category pathophysiological relationships (see Categorical relationships and future directions).

**Figure 2.**
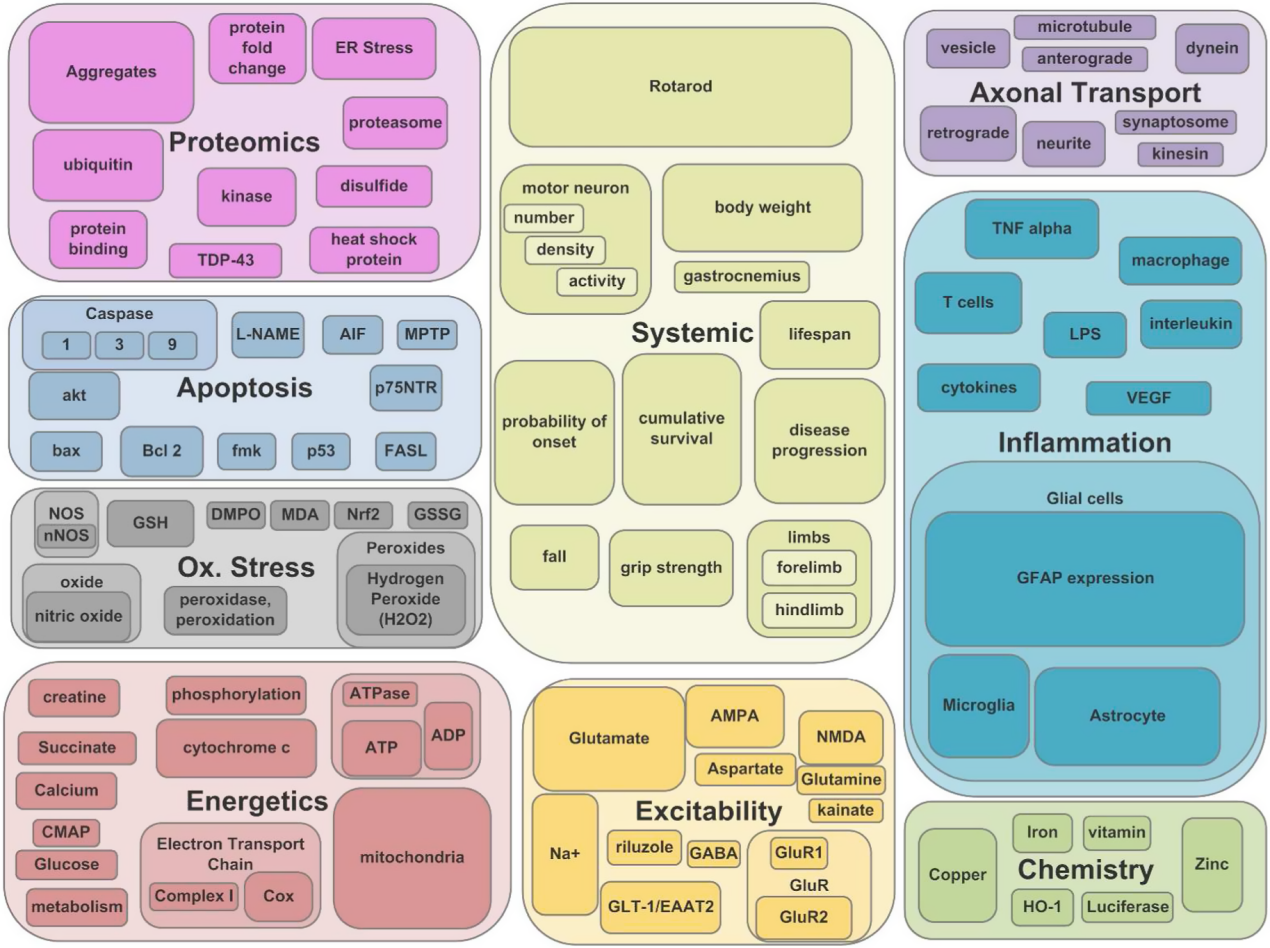
Field map of the most prevalent SOD1-G93A transgenic mouse research topics. The sizes of the boxes represent the relative term frequency. The map illustrates categorical terms required to encompass at least 85% of the articles classified to each of the nine categories: Apoptosis, Axonal transport, Chemistry, Energetics, Excitability, Inflammation, Oxidative stress, Proteomics, and Systemic.

The most common 12 terms of each category (with the variations ‘or’-merged into one search) accounted for the categorization of greater than 85% of the articles in the category. Thus, these most prevalent 12 terms per category were used to develop the field analysis map shown in Figure 2. The field analysis map visually illustrates the proportion of the SOD1-G93A primary data encompassed by each overall category and also the most prevalent within category terms. The size of the boxes corresponds to the approximate relative size of the categories or the categorical terms. In Table I we reveal the number of articles and figures/tables with primary data corresponding to each of the 12 most prevalent terms per category. The full term-category dictionary and field analysis assessment is shown in Supplementary Table I to be found online at http://informahealthcare.com/doi/abs/10.3109/21678421.2015.1047455.

**Table I.** The 12 most common terms or phrases per category are presented with a description and the resultant number of articles (A) and figures (F). Note that the 12 terms provided more than 85% coverage in each category (see Test sets).

Axonal Transport	A	F	Description/Significance
retrograde, retrogradely	44	96	The movement of dynein cargoes towards the cell body
neurite, neurites	38	53	A projection from the cell body, so an axon or dendrite.
dynein	20	84	The motor protein that is responsible for carrying cargoes retrogradely
microtubule, microtubules	17	38	Neural structure or “tracks” on which dynein and kinesin travel.
vesicle	17	38	Transport unit often carried by dynein and kinesin.
anterograde, anterogradely	16	40	The movement of kinesin cargoes from soma towards neuromuscular junction
Synaptosome(s)	11	52	A type of vesicle transported in the axon
axon terminal, projection	10	13	Catch-all terms for detecting axonal transport papers.
kinesin	7	15	The motor protein that is responsible for carrying cargoes anterogradely
loa	7	28	“Legs at Odd Angles” - mutation that affects dynein “legs” (25).
neurofilament* transport	5	16	Neural structure element carried via “slow” axonal transport.
wallerian	4	9	Wallerian Degeneration is the degeneration of an axon.
Chemistry	A	F	Description/Significance
copper, Cu2+	58	236	Used by SOD1
zinc, Zn2+	53	216	Used by SOD1
metal, metals	35	92	Catch-all terms for detecting data discussing metals
luciferase	31	41	An oxidative enzyme used in assays
iron, Fe, Fe2+	17	44	Involved in Fenton reaction, which produces hydroxyl radical
HO-1, Heme oxygenase	17	40	Enzyme that catalyzes the degradation of heme, producing iron
vitamin, B12	16	48	Tried as a treatment (32).
lithium	8	46	Tried as a treatment (33).
ferritin	8	14	Controls iron in a cell
NaHCO3	5	10	Used as a buffer in various experiments, particularly those assessing metalation
Salubrinal	4	11	Used to suppress SOD1 activity (89).
VPA	4	9	Valproic acid. Tried as a treatment (34).
Energetics	A	F	Description/Significance
mitochondrial, mitochondria, mito, mitochondrion,	194	773	Produce ATP, involved in cellular respiration & calcium homeostasis.
calcium, Ca2, Ca2+, ca	100	238	Required for respiration, excitability, and muscle contraction.
Cox, complex IV	80	135	Last enzyme in the respiratory electron transport chain
ATP, ADP, ATPase	74	129	Energy units made or used in cellular respiration
cytochrome, cytochrome c	69	141	Transfers electrons from complex III – IV
phosphorylation	47	89	Process of adding a phosphate group to a protein, turning it “on” or “off”
metabolism, metabolic	43	80	General term encompassing energy harvesting
complex I	28	51	Element of the electron transport chain of mitochondrial respiration
glucose	25	45	Main “fuel” for cellular respiration
creatine	23	75	Increases the formation of ATP
succinate	23	35	Encompasses Succinate Dehydrogenase, aka complex II, role in respiration
depolarization	19	37	Measure of mitochondrial respiration
Excitability	A	F	Description/Significance
Glutamate	108	401	Excess extracellular glutamate causes neuronal degeneration
GLT1, GLT-1, EAAT2, EAAT	56	188	Glutamate transporters; decreases observed in ALS patients and G93A mice
Na, Na+	52	112	Required for action potential; ions enter cell upon activation of glutamate receptors
Excitotoxicity, excitatory,	48	137	Terms describing toxic over-excitation
AMPA	35	107	α-amino-3-hydroxy-5-methyl-4-isoxazole propionic acid- glutamate receptor mediating fast excitatory transmission
cmap, cmaps	35	53	Compound muscle action potential-summation of several action potentials over several muscle fibers in one area
riluzole	33	85	Sodium channel blocker; the only FDA-approved drug for ALS.
NMDA	27	43	N-methyl-D-aspartate- glutamate receptor mediating slow excitatory transmission
GABA	22	50	ϒ-Aminobutyric acid- chief inhibitory neurotransmitter in mammals
GluR*, GluR1, GluR2, GluR	20	60	Glutamate receptors: Down-regulation of GluR2 leads to excess Ca2 + influx
glutamine	16	34	Neurologically inactive form - precursor - of glutamate
aspartate	16	32	Stimulates NMDA receptors, but not as strongly as glutamate does
Oxidative stress	A	F	Description/Significance
H2O2, Hydrogen peroxide	69	129	Free radical. Relatively weak but can produce stronger oxidants.
oxide	47	154	Catch-all term for elements causing oxidation
ROS, reactive oxygen species	47	90	Reactive molecules containing oxygen. Source of free radicals.
GSH, glutathione	45	101	Antioxidant that prevents damage from free radicals.
nitric, nitric oxide	43	145	Also known as NO. Common type of free radical.
Peroxidase, Peroxidation	36	84	Assist in oxidative degradation; activity is enhanced in ALS mice
nNOS, NOS	34	68	Produces NO upon stimulation by inflammatory cytokines
Peroxide, peroxides	26	44	Catch-all term for any peroxide. See hydrogen peroxide.
MDA	15	20	Marker for oxidative stress
Nrf2	15	73	Involved in antioxidant and anti-inflammatory defense (6).
GSSG	14	19	Oxidized form of glutathione
DMPO	11	27	Used in spin trapping to measure the levels of free radicals.
Inflammation	A	F	Description/Significance
GFAP	245	399	Glial fibrillary acidic protein-an indicator of the progression of gliosis
astrocyte	233	579	Astroglia that support neural function; assist in scarring following neural damage
T-cells, T-cell, CD11b, CD4	181	331	Provides neuroprotection
microglia, microglial	172	406	Excess activation leads to neurodegeneration
glial cell, glia	106	247	Catch-all terms for various glial cells.
inflam*, neuroinflamm*, immune	79	285	Catch-all terms for neuroinflammatory processes.
TNF, TNF alpha, TNFalpha, TNFa, TNF a, tumor necrosis factor	66	129	Tumor necrosis factor α. Stimulates immune activation, leading to gliosis
IL, IL 6, IL 4, interleukin	56	78	Interleukins stimulate immune cell activation, leading to gliosis
macrophage, M2, M1	53	88	Regulate immune activity via production of cytokines
VEGF	44	124	Vascular endothelial growth factor. Tried as a treatment (69)
cytokine, cytokines	38	82	Catch-all terms microglial priming and immune cell activation elements
LPS	33	56	Lipopolysaccharide- activates glial cells, inducing gliosis
Apoptosis	A	F	Description/Significance
caspase	122	225	Key apoptotic signal
apoptosis, apoptotic	114	294	Programmed cell death
akt	43	81	When activated by VGEF possibly acts as an anti-apoptotic factor
Bcl 2	31	85	A protein involved in both pro and anti-apoptotic mechanisms
bax	25	43	A pro-apoptotic protein
fmk	19	27	Anti-apoptotic signaling pathway
p75NTR	17	44	p75 neurotrophin receptor promotes caspase-dependent axon degeneration
p53	17	38	Tumor-suppressing protein in apoptosis.
L-NAME	15	28	Reduces NO and reverts the pro-apoptotic factors of it
FASL	11	26	Fas ligand; induces apoptosis when bound to its receptor
MPTP	10	27	Neurotoxin that induces apoptosis
XIAP	9	15	X-linked inhibitor of apoptosis protein
Proteomics	A	F	Description/Significance
Aggregate(s), aggregation,	168	575	Aggregates of mutant, misfolded proteins are a hallmark of ALS
ubiquitin	89	165	Affects protein degradation, trans-location, and interaction
Kinase(s)	65	193	Affects protein activity, signaling; implicated in protein aggregation in ALS
protein binding	58	109	Catch-all term for binding processes of mutant SOD1 leading to aggregation.
proteasome, proteasomal	54	143	Involved in protein degradation.
disulfide	35	131	Disulfide bonds mediate the aggregation process in SOD1
oligomer*	30	40	Catch-all term for proteins
heat shock protein, hsp	30	74	Response to proteomic stress. Up-regulation extends survival.
ER Stress	27	75	Endoplasmic reticulum stress from unfolded proteins
misfolded protein, protein fold change	27	43	The fold change (misfolded proteins) leads to protein aggregates.
protein degradation	24	36	Dysfunctional degradation of misfolded proteins causes aggregates
TDP-43, TDP 43	23	59	TAR-DNA binding protein 43. Inclusions commonly found in ALS patients.
Systemic	A	F	Description/Significance
Density, count or activity of motor neurons (all spellings)	323	483	Includes functional measures of locomotor activity and assessment of motor neuron degeneration
rotarod, rotorod, rot*rod	217	267	Experimental device and test used to assess mouse motor function
disease progression	205	553	Catch-all term for in vivo observation of disease progression.
hindlimb, forelimb, limb,	173	318	Hindlimb tremors are commonly used as a marker of disease onset
body weight	171	233	Indicator of disease progression; decreases in later stages of the disease.
lifespan, life span	121	240	The total time spent alive for the subject.
cumulative survival	103	119	The endpoint of the disease. Total lifespan of subject.
probability of onset	102	109	The time when symptoms of ALS typically begin to appear.
grip, grip strength	94	134	Test assessing mouse's ability to grip; indicator of motor function/progression
in vivo, invivo	88	209	“Within the living” - encompasses all experiments performed on live test subjects.
gastrocnemius	75	151	Large muscle found in the hindlimb, easy to access and evaluate.
fall	59	73	The action of falling down, usually due to inability to stand.

### Topical Analysis within Ontological Categories

We present the informatics results of the topical analysis performed within each of the ontological categories and provide brief explanations of the topics’ significance to the SOD1-G93A transgenic mouse pathophysiology. Given the goals of this informatics-based systematic review, the primary purpose of the following text is to quantify and expound upon the preponderance of articles and sub-topics included in each ontological category. For further in-depth discussion of mechanisms and published experimental study results, we refer the reader to the cited references or topic-specific traditional literature reviews.

*Apoptosis.* Apoptosis, representing 21% of the SOD1-G93A transgenic ALS mouse literature, encompasses all programmed cell death signaling pathways. Apoptosis has multiple relationships with other ontological categories given that the ultimate endpoint of the ALS pathology is cell death.

Caspases, and in particular caspase 1, 3, and 9, are responsible for many of the signaling cascades (13) that initiate apoptosis and, as such, are the most represented term under this category (122 articles, 45% of Apoptosis). Intracerebroventricular administration of zVAD-fmk, a broad caspase inhibitor, has been shown to delay disease onset and mortality in SOD1 ALS mice (13). Other key signals include Bcl-2 (31 articles, 11% of Apoptosis), which has both pro- and anti-apoptotic mechanisms, and Bax (25 articles, 9% of Apoptosis), which is pro-apoptotic (4,14). Many studies have examined the neuroprotective effects of Bcl-2 and how, in abundance, it could be used to abolish the proapoptotic component of Bax in SOD1-G93A mice (4,15).

P53 and p75NTR have been examined equally with 17 articles each, collectively representing 13% of the Apoptosis literature. An increased level of p53 tumor protein is observed in ALS patients (16), but the absence of p53 does not affect the SOD1-G93A mice (17,18). p75NTR is a neurotrophin receptor that regulates signal cascades and functions of cells, and has been implicated in motor neuron degeneration in ALS (19). Reduction of Fas ligands (FASL) was examined in 11 articles as a way to increase survival in ALS mice (20). MPTP, examined in 10 articles, is a neurotoxin known to induce apoptosis that has been shown to increase SOD1 activity when administered to SOD1-G93A mice (21).

*Axonal Transport.* Comprising just 10% of the SOD1-G93A literature, axonal transport has been the least studied pathophysiological category. Molecular motors carry necessary constituents in the axon from the soma to the neuromuscular junction (i.e. anterograde transport via kinesin) and from the neuromuscular junction to the soma (i.e. retrograde transport via dynein) (22,23). Mutations to the machinery and cargoes can impair their attachment to the motor proteins and their mobility (5). Notably, in SOD1-G93A transgenic ALS mice, axonal transport deficits appear well before cell degeneration occurs (24). In addition to possible transport-specific defects, axonal transport is thought to be further hindered due to inadequate mitochondrial ATP (see Energetics), an over-abundance of misfolded SOD protein aggregates (see Proteomics), and possible excitotoxic burdens (see Excitability).

Retrograde movement was the term that appeared most often in the axonal transport literature (44 articles, 23% of Axonal Transport) with dynein ranking third in frequency (20 articles, 14% of Axonal Transport). A mutation in dynein has been shown to rescue axonal transport defects and overall extend the lifespan of ALS SOD1-G93A mice (25). Comparatively, anterograde transport (16 articles, 12% of Axonal Transport) by kinesin (seven articles, 5% of Axonal Transport) does not appear as frequently in the SOD1-G93A literature despite both anterograde and retrograde transport deficits having been documented in SOD1-G93A mice (26).

*Chemistry.* The ontological category of chemistry, accounting for 14% of the SOD1-G93A literature, includes measures of aberrant cellular chemistry, enzymatics, catalytics and metal mishandling present in the SOD1-G93A ALS pathophysiology (2). Copper and zinc collectively represent 39% of the chemistry literature, although their frequency is slightly over-represented due to their appearance in the name of ‘copper zinc superoxide dismutase-1’. Beyond their involvement in SOD1, copper and zinc concentrations have been measured in different locations, with decreases shown in the liver and spinal cord (27). The effect of zinc supplementation in SOD1-G93A mice has been examined, including its increased affect on NMDA-mediated excitotoxicity in SOD1, as well as its negligible impact on survival (28).

Another frequent cellular chemistry assessment is iron homeostasis, which represents 9% of the chemistry literature. Iron homeostasis has been shown to be impaired in both SOD1-G93A transgenic mice and in human ALS patients (29). An increase in iron content and iron genes expression has been observed in G93A-SOD1-transfected neuroblastoma cells compared to wild-type counterparts (6). Iron could also be contributing to the disease via the Fenton reaction, which accelerates hydroxyl radical production that damages cellular DNA (30).

Heme oxygenase-1 (HO-1), representing 9% of the chemistry literature, is an enzyme that assists in degrading heme, and has been observed to increase as ALS progresses (31). Vitamins (32), lithium (33) and valproic acid (VPA) have been explored (34) as treatment options but have shown negligible success.

*Energetics.* Energetics, which encompasses mitochondrial production of ATP via cellular respiration, is the third most represented ontological category, encompassing 39% of the SOD1-G93A transgenic mouse literature. Understandably, mitochondria and variations of this word are the most represented terms, encompassing 39% of the Energetics category itself. As ALS progresses, mitochondrial ability to produce ATP decreases (35), which leads to axonal transport deficiencies, axonal retraction, denervation, and death of cells via apoptosis (36). SOD1-G93A mice mitochondria change in both physical appearance and chemical functionality as the disease progresses (37).

Calcium, the second most prevalent Energetics term, represents 20% of the Energetics ontological category. Calcium overload, a known issue in SOD1-G93A mice, leads to cell death via increased membrane permeability and loss of ATP production. Overexpression of Ca^2+^ binding proteins such as parvalbumin (38) and calbindin D28K (38,39) have been shown to improve disease parameters. Interestingly, most articles examining calcium homeostasis utilize in vitro assessments (40), although in vivo examination is increasing (41). Finally, it should be noted that while calcium is listed in Energetics because the majority of articles examining it investigate calcium handling by mitochondria (42), there are also other aspects of calcium that are clearly related to excitability due to its role in neural transmission and axonal transport (41).

Glucose and related cellular pathway machinery encompasses the remainder of the most prevalent keywords in the Energetics category, as shown in Table I. Specifically, glucose utilization rates are impaired in SOD1-G93A mice in as early as 60 days (43). GAPDH, an enzyme important for the breakdown of glucose for energy, as well as creatine, have also been found to decrease by approximately 40% in SOD1-G93A mouse models (44). Creatine treatment has been shown to protect against excitotoxic lesions created by NMDA (45) and MPTP, which interferes with complex I, slowing mitochondrial metabolism. Finally, complex I, pyruvate, and cytochrome C have all have shown some forms of deficiency in SOD1-G93A mice (35).

*Excitability.* Excitability is ontologically defined as the physiological pathways involved in producing action potentials. Excitability is an integral aspect of the ALS pathophysiology, representing 23% of the SOD1-G93A transgenic mouse literature. More specifically, excitability encompasses excitotoxicity, the pathological toxic over-excitation of neurons that is thought to contribute to the neuronal degeneration seen in SOD1-G93A ALS mice (46). However, there has been recent debate as to whether SOD1-G93A mice experience hyperexcitability (47), hypoexcitability (48) or a combination of both that changes with temporal disease progression (2).

Over-excitation due to glutamate homeostasis is the most frequently cited excitotoxic contributor (160 + articles, 54% of Excitability). Most of the SOD1-G93A specific research has focused on glutamate uptake, concentrations of glutamate in various locations and their effects on the systemic progression of the disease. Since overstimulation is caused by the influx of sodium (56 articles, 18% of Excitability) and calcium ions, various treatments have been tried to inhibit the voltage-gated sodium and calcium channels. Riluzole (12% of Excitability literature), one of the most common treatments for clinical ALS, is thought to work by inactivating the voltage dependent sodium receptors (49,50) on the glutamatergic nerve terminals (46,51).

The two main receptors of glutamate, calcium-permeable AMPA (α-amino-3-hydroxy-5-methyl- 4-isoxazole propionic acid) and NMDA (N-methyl-D-aspartate), are also researched heavily with the SOD1-G93A model and collectively represent 21% of the SOD1-G93A Excitability literature. AMPA receptors lacking GluR2 expression have high calcium permeability and are therefore more susceptible to motor neuron death from excitotoxicity (51). Loss, decrease or immunoreactivity of glutamate transporters (56 articles, 19% of Excitability), including EAAT2 (excitatory amino acid transporter-2) (52), all of which have been shown in SOD1-G93A mice, may also give rise to selective motor neuron degeneration.

GABA, another frequent topic in the excitability literature (7% of Excitability), is a neurotransmitter responsible for regulating excitability. Related topics frequently examined include GABA's current density and amplitude (53), concentration (54), release and transmission upon treatments, by itself (55), or with glycine (56), methionine sulfoximine (MSO) (57), ionomycin (58), and HU210 (59).

Besides glutamate and its related measures, the Compound Muscle Action Potential (CMAP) is the next most frequently investigated measure of excitability (35 articles, 12% of Excitability). While modest at first, it has been observed that CMAP amplitudes drastically decrease in the final weeks before death (60).

In perhaps surprising contrast, other forms of SOD1-G93A traditional electrophysiological properties of motor neurons were not in the top 12 or upper 85% of Excitability terms. However, a sector of research is ongoing with, for example, persistent inward currents or PICs (50,61), frequency-current or F-I gain (48,62), and dendritic processing (63), to name just a few examples.

**Figure 3.**
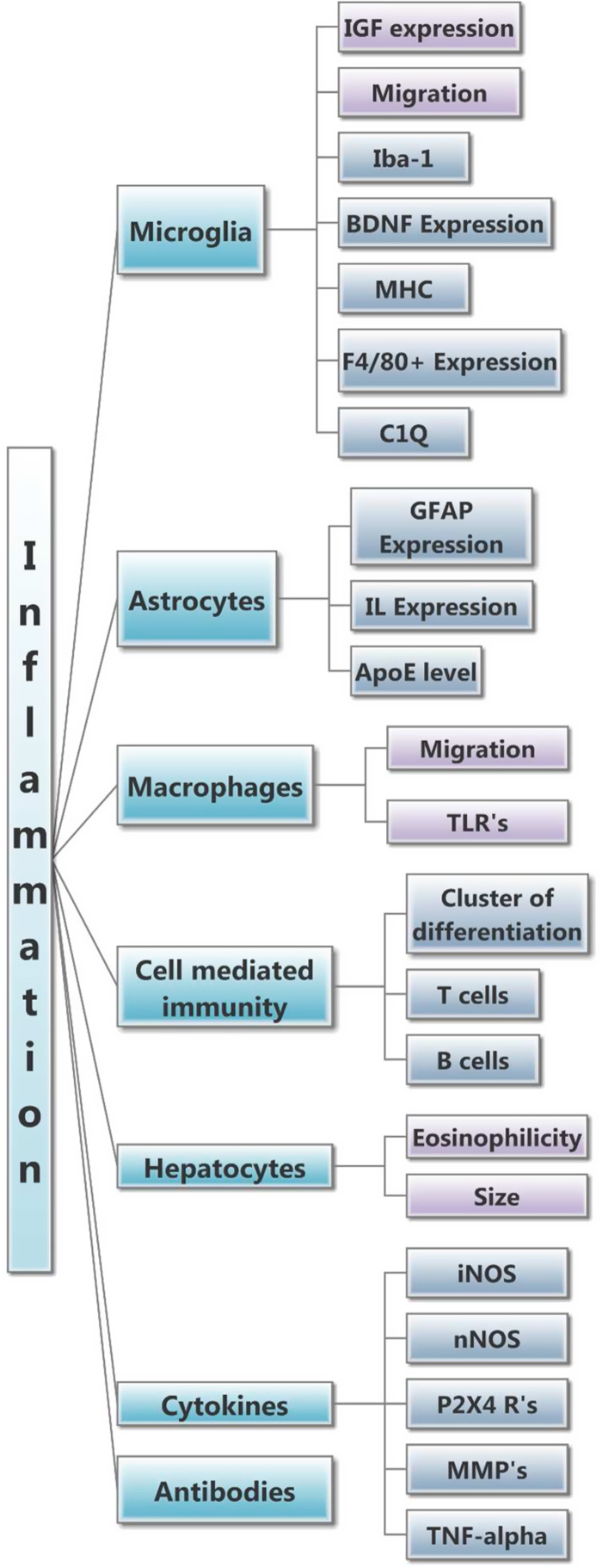
Field map of the inflammation categories based on their pathophysiological properties and significance to demonstrate the variety of terms that were searched. The frequency search reveals that a few measures are used for specific cell types, e.g. GFAP expression was much more heavily used (by 259 articles) compared to ApoE (six articles).

*Inflammation.* One of the fundamental characteristics in progression of ALS is activation of microglia and astrocytes, a process referred to as neuroinflammation. Inflammation is the second-largest SOD1-G93A pathophysiological category as nearly half of the papers were identified as discussing some aspect of neuroinflammation. An in-depth ontological map of this category is shown in Figure 3. A major goal of inflammation research is to determine which parameters are expediting the disease versus which ones are protecting against it.

The degree of gliosis, scarring caused by reactive astrocytes, is a major indication of ALS progression in SOD1-G93A mice (64), as such reactive inflammatory cells are thought to contribute to death of the motor neurons (MNs). Not surprisingly, nearly half of the most prevalent inflammation key terms is related to some aspect of gliosis. Gliosis is often assessed using GFAP expression (64), which is also the most prevalent key word in the inflammation literature (240 + articles, 39% of Inflammation).

Microglial activation was the second-most assessed inflammatory topic (28% of Inflammation). In particular, common direct measures encompassed cytokines, including iNOS, TNF-alpha, and various interleukins (IL). An increased level of TNF-alpha (11% of Inflammation) has been observed in human ALS patients, but the absence of TNF-alpha in SOD1-G93A mice did not affect the survival (65). Nonetheless, lowering the activity of other cytokines, such as IL-1beta, has been shown to reduce inflammation and extend survival in SOD1-G93A mice (66). Additionally, macrophage activation (9% of Inflammation), which results in the production of cytokines, has been investigated in SOD1-G93A mice and in clinical patients, where an up-regulation is commonly seen (66). Therefore, unsurprisingly, migration of microglia and macrophages is often used to evaluate the effectiveness of anti-inflammatory treatments (67).

Vascular endothelial growth factor (VEGF), which has been investigated in 44 articles (7% of Inflammation), plays a role in neuronal protection from ischemic and hypoxic damages (68), and, used as a treatment, has shown promising results in both SOD1-G93A mice and in humans (69). Although VEGF has been classified under Inflammation due to its protective effects on neuroinflammation, studies suggest it may also act to reduce excitotoxicity and downstream apoptotic pathways (68).

*Oxidative Stress.* Oxidative stress, representing 19% of the SOD1-G93A literature, reflects an imbalance between the systemic manifestation of reactive oxygen species and the normal physiological ability to readily detoxify the reactive intermediates or to repair the resulting damage (30). Disturbances in the normal redox state of cells, such as those in SOD1-G93A transgenic mice, can cause toxic effects through the production of peroxides and free radicals. Resulting damage can affect many components of the neural and glial cells, including proteins, lipids, and DNA (9). Furthermore, some reactive oxidative species act as cellular messengers in redox signaling. Thus, oxidative stress can interfere with normal mechanisms of cellular signaling, and so many oxidative stress articles also examine their effects on other ontological categories, including excitability, inflammation and systemic outcomes.

Peroxides, and specifically hydrogen peroxide (H_2_O_2_), represent 29% of the SOD1-G93A oxidative stress literature. Peroxides are produced during the electron leakage from mitochondria (9). Thus, most such articles focus on reducing the effects of peroxides produced by damaged mitochondria (70,71).

Other free radicals and oxidants for which the effects of various treatments have been investigated include nitric oxide (NO-) (72) and peroxynitrite (ONOO-) (73). Transcription factor Nrf2 (6% of Oxidative stress) is known to interact with the antioxidant-response element enhancer sequence to increase protein expression involved in antioxidant defense (74). In the SOD1-G93A model, a significant decrease has been cited in the expressions of antioxidant response genes regulated by Nrf2 (75) and the effects of the Nrf2/ARE system activation (76). Other highly cited antioxidants include glutathione and peroxidase, which were found in over 75 + SOD1-G93A articles (33% of Oxidative stress).

*Proteomics.* The cellular stress caused by aggregates of mutant, misfolded proteins is a hallmark of neurodegenerative diseases including ALS (36). The misfolded and aggregated proteins are considered to play a lead role in the pathophysiology of the SOD1-G93A transgenic ALS mouse. Hence, the majority of papers in proteomics, 168 or 40% of all proteomics-labeled articles, were concerned with aggregation. Mutant SOD1 seems to impair the proteasomal pathway and autophagy of the cell's degradation machinery (36). Thus, instead of undergoing degradation, these misfolded proteins begin to aggregate in the cell. Like the amyloid tangles in Alzheimer's disease, a major question of the proteomics field has been whether these misfolded aggregates are a cause of the ALS pathology, in and of themselves, or if they are simply a pathological side-effect (77).

Misfolded protein degradation is dependent on proteasomes (54 papers, 13% of Proteomics), which have been shown to be impaired in SOD1-G93A mice (78). The two most common proteasomes studied are LMP2 and LMP7, mentioned by eight and six papers, respectively (Supplementary Table I to be found online at http://informahealthcare.com/doi/abs/10.3109/21678421.2015.1047455). Ubiquitin (89 articles, 21% of Proteomics) labels proteins for degradation, and has also been shown to be inappropriately included in aggregates (10).

Many patients with ALS show inclusions containing ubiquitinated and phosphorylated TAR-DNA binding protein 43 (36). Most of the articles, 23 in total, examined the amount of TDP-43 as a measure of proteomic progression in SOD1-G93A ALS mice. Additional stress factors resulting from protein aggregation, such as overexpression of various heat shock proteins (HSP) and ER stress (54 articles, 13% of Proteomics), are increased in SOD1-G93A mice and are thought to coincide with disease progression (79). The effect of disulfide-linking at cysteine sites has been shown to slow the rate of mutant SOD1 degradation (80) and subsequently increase aggregation (81).

*Systemic.* The systemic category includes measures that examine the disease on a higher physiological scale including overall tissue death, functional outcomes, and other possible contributors of non-neuromuscular origin. Additionally, disease onset and endpoint measures are contained within the systemic category. Systemic evaluations are important to SOD1-G93A research because they demonstrate the point at which the pathology is impacting overall function and/or health. Thus, it is not surprising that the systemic category is the most frequently assessed SOD1-G93A ontological category (see Figure 1). Many articles utilize systemic measures to assess their possible relationship to primary experimental measures from other ontological categories.

Rotarod performance (a motor function test where the mouse is placed on a rod rotating at either a constant or accelerating speed and time is measured until the animal falls), grip strength, and grip endurance, are all in vivo physical motor function tests that are utilized to assess neuromuscular disease progression in transgenic SOD1-G93A ALS mice (3). Collectively, these functional measures account for approximately 28% of the systemic category.

Neuronal density and overall motor neuron count, which are known to continuously decrease with SOD1-G93A ALS mouse disease progression, are the most prevalent measures of the systemic category, encompassing 41% of the systemic literature. Unlike the functional measures, they can be measured in either in vivo or in vitro experimental settings.

Systemic measures are also used to assess the disease onset and endpoint in in vivo experiments with SOD1-G93A transgenic ALS mice. Measures of onset appear in greater than 98% of articles assigned to the systemic category. Onset is typically defined by the start of a wobbly gait, hindlimb clasping, or a percent drop in rotarod performance (82). The endpoint is typically defined as either full limb paralysis, failure to stand on a rotating rotarod, or death (3,79).

The least represented sector of the systemic category is articles examining potential disease contributors of non-neuromuscular origin. Only a handful of articles were found. Examples include the role of possible liver disease (83) and T-lymphocytes (84). More research is needed in this non-neuromuscular sector given recent clinical findings citing the high relevance and relationships to overall health and/or other antecedent disease (85).

### Categorical relationships and future directions

ALS has long been considered a multi-factorial disease (86). This characterization is supported by the many biological and pathological connections between the different presented ontological categories of the SOD1-G93A ALS pathophysiology. Hence, it is not surprising that each primary data article is typically represented by 2–3 different ontological categories. For instance, excitability (electrophysiology, channels, neurotransmitters, etc.) cannot be adequately studied without considering its strong ties to energetics (ATP, mitochondrial calcium homeostasis, etc.) and axonal transport (transport of mitochondria and neurotransmitters, etc.). A further complication of such pathophysiological relationships is that they do not necessarily have the same sign or direction for the entire disease duration. For example, axonal transport appears to initially increase, possibly as a compensatory mechanism, prior to later showing deficits (2,5,87). Experimental measurement of relationships, especially temporal cross-category relationships (e.g. relationship of excitotoxicity to energetics) is difficult due to the required longitudinal and combinatorial experimental design (2,88)

Temporal relationships in the high-copy mouse model have already been shown to drastically impact the overall dynamics of the SOD1-G93A ALS pathophysiology (2,48). Recent clinical ALS evidence has suggested possible neuroprotective effects related to pre-onset homeostatic regulation and possibly even hypervigilant regulation (85). Additionally, theoretical analysis has shown that treatments which address underlying system-level mathematical regulatory instabilities, including homeostatic oscillations, are more promising than traditional single-mechanism strategies (2,23). Collectively, current evidence indicates that future experimental and informatics analysis of within-category and cross-category SOD1-G93A pathophysiological temporal relationships is needed to help solve the many remaining mysteries.

## Supplementary material available online

Supplementary Table I to be found online at http://informahealthcare.com/doi/abs/10.3109/21678421.2015.1047455.
