# Supplementary material for: State of the field: An informatics-based systematic review of the SOD1-G93A amyotrophic lateral sclerosis transgenic mouse model
Source: Amyotroph Lateral Scler Frontotemporal Degener. 2015 May 22;17(1-2):1–14. doi: 10.3109/21678421.2015.1047455 (PMC4724331; doi:10.3109/21678421.2015.1047455)
Supplement: Supplementary file 1 [file iafd_a_1047455_sm3784.pdf]

Supplementary material for Kim R B. et al. State of the field: An informatics-based systematic review of the SOD1-G93A amyotrophic lateral sclerosis transgenic mouse model. Amyotroph Lateral Scler Frontotemporal Degener. 2015;doi:10.3109/21678421.2015.1047455.

Supplementary Table 1: This term-category dictionary lists the entire list of terms used in the search, along with its ontological category and the resultant number of identified figures (F) and the corresponding overall articles (A). The "Uncategorized" represent the terms that were initially included in the search but later disregarded due to too high a false positive rate.

Keyword	A	F	Ontology
motor	689	2440	Uncategorized
protein	617	1668	Uncategorized
NO	551	1019	Uncategorized
survival	517	1283	Uncategorized
onset	388	856	Uncategorized
antibody	311	659	Uncategorized
weight	306	448	Uncategorized
superoxide	262	1330	Uncategorized
gene	253	743	Genetic Damage
proteins	247	563	Uncategorized
GFAP	245	399	Inflammation
actin	234	392	Uncategorized
muscle	217	603	Uncategorized
rot*rod	217	267	Systemic
rotarod	211	261	Systemic
immunoreactivity	206	463	Uncategorized
disease progression	205	553	Systemic
astrocytes	204	509	Inflammation
antibodies	200	396	Uncategorized
motor neuron* number	189	239	Systemic
body weight	171	233	Systemic
mitochondrial	158	611	Energetics
glial	154	375	Uncategorized
fold	147	239	Genetic Damage
mitochondria	144	411	Energetics
microglia	134	313	Inflammation
CASP*	129	241	Apoptosis
Cu	124	530	Uncategorized
T cells	123	218	Inflammation
aggregates	123	270	Proteomics
caspase	122	225	Apoptosis
Zn	119	531	Uncategorized
axons	115	201	Uncategorized
Glutamate	108	401	Excitability
astrocyte	105	186	Inflammation
cumulative survival	103	119	Systemic
immunoreactive	102	166	Uncategorized
Probability of onset	102	109	Systemic
microglial	100	196	Inflammation
rna	97	172	Genetic Damage
enzyme	97	159	Uncategorized
oxidative	95	275	Uncategorized
ChAT	94	160	Uncategorized
grip	94	134	Systemic
motoneuron	90	322	Uncategorized
muscles	90	232	Uncategorized
caspase 3	90	163	Apoptosis
T cell	89	144	Inflammation
ubiquitin	89	165	Proteomics
hindlimb	89	149	Systemic
in vivo	88	209	Systemic
gapdh	86	143	Uncategorized
aggregation	86	336	Proteomics
cell viability	85	132	Uncategorized
DAPI	85	133	Uncategorized
genes	83	251	Genetic Damage
axonal	79	236	Uncategorized
gastrocnemius	75	151	Systemic
neurofilament	74	139	Uncategorized
apoptosis	71	206	Apoptosis
lifespan	71	136	Systemic
overexpression	70	216	Genetic Damage
apoptotic	70	126	Apoptosis
glial cells	70	142	Inflammation
cytochrome	69	141	Energetics
NeuN	69	90	Uncategorized
motoneuron* number	67	95	Systemic
Cox	66	104	Energetics
cytochrome c	64	128	Energetics
EGFP	64	161	Uncategorized
limb	63	113	Systemic
H2O2	62	110	Oxidative Stress
life span	62	115	Systemic
kinase	61	188	Proteomics
TNF	59	116	Inflammation
fall	59	73	Systemic
protein binding	58	109	Proteomics
copper	56	233	Chemistry
ATP	55	80	Energetics
motor activity	55	90	Systemic
calcium	53	141	Excitability
IL	53	74	Inflammation
zinc	50	211	Chemistry
Ca2	50	118	Excitability
synthase	50	145	Uncategorized
mns	50	146	Uncategorized
MTT	50	82	Uncategorized
Na	50	112	Excitability
Ca2+	49	116	Excitability
ER	49	131	Uncategorized
proteasome	49	134	Proteomics
glial cell	49	93	Inflammation
inflam*	49	156	Inflammation
fold change	48	87	Genetic Damage
phosphorylation	47	89	Energetics
oxide	47	154	Oxidative Stress
caspase 1	46	85	Apoptosis
oxygen	45	90	Uncategorized
CD11b	45	65	Inflammation
TNF alpha	44	93	Inflammation
vegf	44	124	Inflammation
nitric	43	145	Oxidative Stress
akt	43	81	Apoptosis
nitric oxide	42	144	Oxidative Stress
oxidase	42	92	Uncategorized
pbs	42	101	Uncategorized
ROS	40	66	Oxidative Stress
aggregate	39	96	Proteomics
mortality	39	62	Systemic
Glutathione	37	89	Oxidative Stress
oxidation	37	67	Uncategorized
limbs	37	44	Systemic
reflex	37	49	Systemic
igf	36	117	Uncategorized
IGF	36	117	Uncategorized
cmap	35	51	Excitability
ampa	35	107	Excitability
TNF a	35	65	Inflammation
gdnf	35	102	Uncategorized
disulfide	35	131	Proteomics
ca	34	43	Excitability
Stride	34	49	Systemic
retrograde	33	76	Axonal Transport
Iba 1	33	44	Inflammation
LPS	33	56	Inflammation
riluzole	32	85	Excitability
sirna	32	51	Genetic Damage
Inflammatory	32	86	Inflammation
locomotor	32	46	Systemic

step	32	56	Systemic
caspase 2	32	51	Apoptosis
antioxidant	31	81	Uncategorized
Bcl 2	31	85	Apoptosis
cleaved caspase 3	31	47	Apoptosis
luciferase	31	41	Chemistry
enzymatic	31	40	Uncategorized
stem cells	31	104	Inflammation
metal	31	86	Chemistry
hydrogen	30	48	Uncategorized
glia	30	66	Inflammation
forelimb	30	61	Systemic
paw	30	60	Systemic
oligomer*	30	40	Proteomics
glt	29	82	Excitability
iNOS	29	57	Inflammation
fold changes	29	36	Genetic Damage
edl	29	77	Systemic
motorneurons	29	40	Uncategorized
running	29	38	Systemic
muscle denervation	29	61	Systemic
caspase 10	29	33	Apoptosis
complex I	28	51	Energetics
cytokine	28	50	Inflammation
gait	28	62	Systemic
motor neuron* density	28	33	Systemic
dendrites	27	49	Uncategorized
nmda	27	43	Excitability
ER Stress	27	75	Proteomics
dmso	27	48	Uncategorized
metabolism	26	48	Energetics
trigeminal	26	34	Systemic
muscle strength	26	34	Systemic
caspase 8	26	36	Apoptosis
neurofilaments	25	47	Uncategorized
glucose	25	45	Energetics
mito	25	64	Energetics
ldh	25	29	Uncategorized
peroxide	25	43	Oxidative Stress
cytokines	25	49	Inflammation
IL 6	25	33	Inflammation
immune	25	73	Inflammation
bax	25	43	Apoptosis
distal	25	43	Systemic
caspase 9	25	29	Apoptosis
gsh	24	46	Oxidative Stress
tdp	24	62	Genetic Damage
antigen	24	41	Uncategorized
IL 4	24	25	Inflammation
protein degradation	24	36	Proteomics
GLT-1	24	70	Excitability
neurite	23	32	Axonal Transport
creatine	23	75	Energetics
Excitotoxicity	23	83	Excitability
qPCR	23	36	Genetic Damage
M2	23	33	Inflammation
TDP 43	23	59	Proteomics
TDP-43	23	59	Proteomics
stem cell	23	72	Inflammation
misfolded protein*	23	50	Proteomics
motor neuron* count	23	25	Systemic
neurites	22	28	Axonal Transport
ADP	22	37	Energetics
EAAT2	22	72	Excitability
gaba	22	50	Excitability
succinate	22	35	Energetics
tyrosine	22	30	Proteomics
enzymes	22	31	Uncategorized
Na+	22	55	Excitability
hydrogen peroxide	22	39	Oxidative Stress

caspase 6	22	32	Apoptosis
insulin	21	68	Uncategorized
peroxidase	21	34	Oxidative Stress
Heat shock protein	21	58	Proteomics
dynein	20	84	Axonal Transport
excitatory	20	35	Excitability
nNOS	20	47	Oxidative Stress
gliosis	20	25	Inflammation
endurance	20	39	Systemic
GluR*	20	60	Excitability
complex IV	20	36	Energetics
depolarization	19	37	Energetics
KD	19	24	Energetics
nos	19	27	Oxidative Stress
Inflammation	19	75	Inflammation
myelin	19	29	Inflammation
fmk	19	27	Apoptosis
reactive oxygen species	19	41	Oxidative Stress
caspase 7	19	21	Apoptosis
metabolic	18	32	Energetics
macrophage	18	24	Inflammation
hindlimbs	18	34	Systemic
hindlimbs	18	34	Systemic
retrogradely	18	26	Axonal Transport
vesicle	17	38	Axonal Transport
atpase	17	30	Energetics
complex II	17	30	Energetics
Rotenone	17	21	Energetics
acetylcholine	17	24	Uncategorized
peroxidation	17	50	Oxidative Stress
BDNF	17	31	Uncategorized
bone marrow	17	52	Inflammation
p53	17	38	Apoptosis
amyloid	17	47	Proteomics
forelimbs	17	20	Systemic
p75NTR	17	44	Apoptosis
neuroinflamm*	17	72	Inflammation
complex III	17	24	Energetics
dendrite	16	24	Uncategorized
aspartate	16	32	Excitability
GLT1	16	50	Excitability
glutamine	16	34	Excitability
IFN	16	23	Inflammation
malate	16	44	Uncategorized
NADPH	16	32	Energetics
quadriceps	16	29	Systemic
HO-1	16	31	Chemistry
anterograde	15	38	Axonal Transport
BrdU	15	18	Energetics
hypoxia	15	39	Energetics
pyruvate	15	26	Energetics
kainate	15	37	Excitability
mda	15	20	Oxidative Stress
Nrf2	15	73	Oxidative Stress
ccs	15	46	Genetic Damage
p50	15	24	Genetic Damage
macrophages	15	29	Inflammation
L-NAME	15	28	Apoptosis
pdi	15	38	Proteomics
mune	15	38	Systemic
neuron density	15	15	Systemic
Microtubule	14	30	Axonal Transport
citrate	14	21	Energetics
gssg	14	19	Oxidative Stress
NADH	14	20	Energetics
proteasomal	14	23	Proteomics
rotorod	14	14	Systemic
catalytic	14	20	Uncategorized
oxidative damage	14	31	Uncategorized
GluR2	14	37	Excitability

vitamin	13	42	Chemistry
Lymphocytes	13	35	Inflammation
M1	13	21	Inflammation
T beta	13	15	Inflammation
tumor necrosis factor	13	33	Inflammation
erk	13	22	Proteomics
synuclein	13	23	Proteomics
dhk	12	19	Excitability
Ib	12	18	Uncategorized
NF kB	12	16	Inflammation
NG2	12	28	Inflammation
TGF	12	16	Inflammation
chymotrypsin	12	21	Proteomics
protein fold change	12	17	Proteomics
hsp	12	23	Proteomics
mapk	12	17	Proteomics
digitorum	12	18	Systemic
muscle weakness	12	20	Systemic
synaptosomes	11	51	Axonal Transport
iron	11	34	Chemistry
hypoxic	11	36	Energetics
excitotoxic	11	26	Excitability
dmpo	11	27	Oxidative Stress
paraquat	11	25	Oxidative Stress
peroxynitrite	11	22	Oxidative Stress
CD4	11	20	Inflammation
interleukin	11	15	Inflammation
FASL	11	26	Apoptosis
MMP	11	34	Proteomics
rostral	11	17	Uncategorized
li	11	23	Uncategorized
Bcl xL	11	29	Energetics
FCCP	10	22	Energetics
ant	10	19	Genetic Damage
adenovirus	10	21	Inflammation
chemokine	10	15	Inflammation
mononuclear	10	26	Inflammation
mptp	10	27	Apoptosis
Olig2	10	20	Inflammation
axon terminal	9	12	Axonal Transport
cmaps	9	12	Excitability
metabolite	9	25	Energetics
sdh	9	14	Energetics
pdc	9	14	Excitability
NOX	9	17	Oxidative Stress
NOX2	9	15	Oxidative Stress
OH	9	19	Oxidative Stress
ttc	9	36	Genetic Damage
DC	9	9	Inflammation
F4/80	9	14	Inflammation
mcp	9	24	Inflammation
monocytes	9	36	Inflammation
cleaved parp	9	10	Apoptosis
par	9	14	Apoptosis
xiap	9	15	Apoptosis
bv	9	19	Proteomics
kinases	9	14	Proteomics
exercise	9	24	Systemic
forces	9	20	Systemic
locomotion	9	26	Systemic
swimming	9	11	Systemic
track	9	10	Systemic
metals	9	9	Chemistry
GluR1	9	21	Excitability
Lithium	8	46	Chemistry
CO2	8	16	Uncategorized
cycloheximide	8	12	Energetics
hif	8	22	Energetics
AIF	8	19	Apoptosis
bso	8	10	Excitability

peroxisome	8	34	Oxidative Stress
cgrp	8	13	Genetic Damage
eosinophilic	8	20	Inflammation
Lymphocyte	8	10	Inflammation
MHC	8	13	Inflammation
monocyte	8	18	Inflammation
zvad	8	12	Apoptosis
LMP2	8	13	Proteomics
proteomic	8	39	Proteomics
body condition	8	15	Systemic
ferritin	8	14	Chemistry
kinesin	7	15	Axonal Transport
loa	7	28	Axonal Transport
cytosolic calcium	7	10	Excitability
mitochondrion	7	8	Energetics
parvalbumin	7	31	Energetics
tcep	7	17	Excitability
GCLM	7	18	Oxidative Stress
C1Q	7	7	Inflammation
fgf	7	24	Inflammation
G CSF	7	21	Inflammation
interferon	7	19	Inflammation
NAD	7	14	Energetics
PGE2	7	12	Inflammation
pioglitazone	7	23	Uncategorized
pi3	7	13	Apoptosis
food intake	7	8	Systemic
plantar	7	16	Systemic
triceps	7	19	Systemic
hMSC	7	24	Inflammation
Antimycin A	6	9	Energetics
EA	6	20	Energetics
glycogen	6	18	Energetics
NT immunoreactivity	6	13	Energetics
PMN	6	16	Systemic
rhod	6	9	Energetics
pna	6	14	Excitability
apocynin	6	13	Inflammation
ApoE	6	12	Inflammation
CD11c	6	7	Inflammation
CD8	6	6	Inflammation
myeloid	6	13	Inflammation
rapamycin	6	23	Inflammation
casp	6	8	Apoptosis
pba	6	13	Apoptosis
pgsk	6	6	Apoptosis
arimoclomol	6	15	Proteomics
LMP7	6	13	Proteomics
tongue	6	11	Systemic
MSC	6	20	Inflammation
Fe	5	9	Chemistry
NaHCO3	5	10	Chemistry
Zn2+	5	11	Chemistry
CCCP	5	5	Energetics
dtpa	5	11	Excitability
GCLC	5	11	Oxidative Stress
atf	5	8	Genetic Damage
Log2	5	8	Genetic Damage
GRP	5	13	Inflammation
ifngamma	5	16	Inflammation
Ki67	5	9	Inflammation
rofecoxib	5	11	Inflammation
splenocytes	5	5	Inflammation
tnfalpha	5	10	Inflammation
white blood	5	5	Inflammation
kdel	5	11	Proteomics
MMP9	5	8	Proteomics
general condition	5	5	Systemic
reflexes	5	7	Systemic
copper content	5	8	Chemistry

copper effect	5	11	Chemistry
zinc treatment	5	17	Chemistry
hMSCs	5	18	Inflammation
motoneuron* density	5	5	Systemic
coenzyme*	5	5	Chemistry
neurofilament* transport	5	16	Axonal Transport
synaptosome	4	6	Axonal Transport
wallerian	4	9	Axonal Transport
Cu2+	4	4	Chemistry
Salubrinal	4	11	Chemistry
vpa	4	9	Chemistry
caffeine	4	8	Uncategorized
carnitine	4	13	Energetics
geldanamycin	4	4	Energetics
cellular respiratory	4	5	Energetics
ubiquinone	4	6	Energetics
ACh	4	5	Excitability
ahp	4	8	Excitability
dhpg	4	11	Excitability
eaat	4	6	Excitability
glur	4	12	Excitability
ivermectin	4	11	Excitability
memantine	4	7	Excitability
voltage threshold	4	6	Excitability
ndga	4	13	Oxidative Stress
O2-	4	8	Oxidative Stress
ONOO-	4	4	Oxidative Stress
ppar	4	11	Oxidative Stress
Cxcr4	4	14	Genetic Damage
CCL2	4	5	Inflammation
celecoxib	4	11	Inflammation
CX3CR1	4	11	Inflammation
granulocyte	4	22	Inflammation
hepatocyte	4	12	Inflammation
leukocyte	4	4	Inflammation
leukocytes	4	6	Inflammation
neutrophils	4	4	Inflammation
dorfin	4	18	Proteomics
ntr	4	7	Apoptosis
erp57	4	6	Proteomics
survivors	4	4	Systemic
MSCs	4	10	Inflammation
anterogradely	4	4	Axonal Transport
Heme oxygenase	4	14	Chemistry
motoneuron* count	4	4	Systemic
peroxides	4	4	Oxidative Stress
B12	3	6	Chemistry
bicarbonate	3	11	Chemistry
Fe2+	3	3	Chemistry
breathing	3	9	Energetics
BzATP	3	7	Energetics
epoxomicin	3	6	Energetics
uridine	3	16	Energetics
ventilation	3	5	Energetics
egcg	3	12	Excitability
mglur	3	3	Excitability
mso	3	15	Excitability
peroxisomes	3	12	Oxidative Stress
ppargamma	3	17	Oxidative Stress
Hcy	3	4	Genetic Damage
p47	3	4	Genetic Damage
rag	3	3	Genetic Damage
tardbp	3	16	Genetic Damage
adnf	3	11	Inflammation
C3b	3	4	Inflammation
CCR2	3	10	Inflammation
hepatocytes	3	9	Inflammation
lenalidomide	3	14	Inflammation
LY6C	3	12	Inflammation
mnc	3	16	Inflammation
pio	3	8	Inflammation
thalidomide	3	10	Inflammation
TIP	3	3	Inflammation
vegfa	3	5	Inflammation
WBC	3	3	Inflammation
aggregating	3	3	Proteomics
disulfide linking	3	11	Proteomics
S9	3	3	Proteomics
tyr	3	6	Proteomics
grps	3	12	Systemic
copper treatment	3	8	Chemistry
zinc content	3	4	Chemistry
zinc effect	3	9	Chemistry
folic	2	7	Chemistry
Folic Acid	2	7	Chemistry
8OH2'dG	2	3	Energetics
BBG	2	3	Energetics
caprylic	2	6	Energetics
carbon dioxide	2	2	Energetics
coenzyme q10	2	2	Energetics
cystine	2	9	Energetics
keto	2	2	Energetics
PPX	2	8	Energetics
riboflavin	2	4	Energetics
triglyceride	2	6	Energetics
ubiquinol	2	3	Energetics
UCP3	2	2	Energetics
CoCL2	2	5	Energetics
ini	2	7	Excitability
nbqx	2	3	Excitability
nrf	2	3	Excitability
rpr	2	10	Excitability
antioxidative	2	17	Uncategorized
T BHQ	2	2	Oxidative Stress
mlgf	2	5	Genetic Damage
napb	2	4	Genetic Damage
psa	2	5	Genetic Damage
aml	2	3	Inflammation
antigens	2	2	Uncategorized
bmdm	2	4	Inflammation
CD206	2	4	Inflammation
CD25	2	2	Inflammation
Eosinophils	2	2	Inflammation
FGFR 1	2	2	Inflammation
lymph	2	2	Inflammation
neutrophil	2	2	Inflammation
P2X4	2	15	Inflammation
splenocyte	2	2	Inflammation
TCR	2	7	Inflammation
TH1	2	2	Inflammation
toll like	2	3	Inflammation
bee venom	2	8	Uncategorized
LMP	2	2	Proteomics
prp	2	7	Proteomics
ambulatory	2	3	Systemic
functional rating	2	2	Systemic
mastication	2	5	Systemic
mstn	2	2	Systemic
nbp	2	11	Systemic
swallow	2	6	Systemic
DHBA	2	4	Oxidative Stress
GLP-1	2	8	Energetics
fEPSP	2	2	Excitability
^{125}I-LIF	1	1	Axonal Transport
hc555	1	3	Axonal Transport
hydroxytyptophan	1	1	Axonal Transport
KAP3	1	8	Axonal Transport
musk	1	4	Axonal Transport
MuSK L	1	4	Axonal Transport
MuSK-L	1	4	Axonal Transport

APYrase	1	2	Energetics
Caprylic triglyceride	1	5	Energetics
coq10	1	5	Energetics
DCA	1	7	Energetics
DP 109	1	7	Energetics
DP 460	1	7	Energetics
DP 460	1	7	Energetics
Energetics	1	1	Energetics
metformin	1	7	Energetics
NHOH	1	2	Energetics
phenylhydrazone	1	1	Energetics
cellular respiration	1	1	Energetics
SS 31	1	4	Energetics
cddo	1	10	Excitability
cycad	1	1	Excitability
epsp	1	1	Excitability
glutamte	1	1	Excitability
anti-oxidant	1	2	Uncategorized
genistein	1	1	Oxidative Stress
superoxides	1	1	Oxidative Stress
Homocisteine	1	1	Genetic Damage
mapt	1	1	Genetic Damage
npepps	1	3	Genetic Damage
CD 25	1	1	Inflammation
CD 4	1	1	Inflammation
CD169	1	1	Inflammation
celcoxib	1	1	Inflammation
DCs	1	1	Inflammation
diapocynin	1	7	Inflammation
FoxP3	1	1	Inflammation
granulocytes	1	1	Inflammation
hgrp	1	9	Inflammation
igfbp	1	1	Inflammation
IL1R	1	1	Inflammation
MAFB	1	1	Inflammation
MDSC	1	1	Inflammation
MDSCs	1	1	Inflammation
NADP(H)	1	1	Energetics
NKT	1	6	Inflammation
osteopontin	1	1	Inflammation
p trk	1	4	Inflammation
SAM on motor performance	1	1	Inflammation
sulindac	1	5	Inflammation
teff	1	3	Inflammation
TLR	1	1	Inflammation
TLRs	1	2	Inflammation
treg	1	3	Inflammation
Ym	1	1	Inflammation
c abl	1	8	Apoptosis
C-abl	1	8	Apoptosis
colivelin	1	3	Apoptosis
dasatinib	1	4	Apoptosis
dihydropyrimidinase	1	3	Proteomics
kdelr	1	6	Proteomics
MMPs	1	1	Proteomics
gwt	1	1	Systemic
hnsc	1	5	Systemic
psnl	1	7	Systemic
catalysis	1	1	Uncategorized
catalyst	1	1	Uncategorized
Ex-4	1	6	Energetics
[Zn]	1	1	Chemistry
axonal projection	1	1	Axonal Transport

NOX4	1	1	Oxidative Stress
chromium	0	0	Chemistry
citruline	0	0	Chemistry
Salubrine	0	0	Chemistry
coenzyme q	0	0	Energetics
coq	0	0	Energetics
corticosteroid	0	0	Energetics
corticosteroids	0	0	Energetics
egb	0	0	Energetics
energetic	0	0	Energetics
epoxomycin	0	0	Energetics
ETC (enyzmatic activities)	0	0	Energetics
FADH	0	0	Energetics
fumarate	0	0	Energetics
gluconeogenesis	0	0	Energetics
Maleate	0	0	Energetics
oxaloacetate	0	0	Energetics
phosphotase	0	0	Energetics
purinaceptor	0	0	Energetics
SS31	0	0	Energetics
ubidecarenone	0	0	Energetics
tertbutyl hydroperoxide	0	0	Oxidative Stress
gene silencer	0	0	Genetic Damage
STAT6	0	0	Genetic Damage
CCL11	0	0	Inflammation
CD 11b	0	0	Inflammation
CD 206	0	0	Inflammation
CD 8	0	0	Inflammation
CD103	0	0	Inflammation
CX3C	0	0	Inflammation
Eosinophil	0	0	Inflammation
eosinophilia	0	0	Inflammation
LMIIA-24	0	0	Apoptosis
MAF	0	0	Inflammation
nimesulfide	0	0	Inflammation
NLR	0	0	Inflammation
NLRs	0	0	Inflammation
OPN	0	0	Inflammation
opsonin	0	0	Inflammation
phagocyte	0	0	Inflammation
phagocytes	0	0	Inflammation
PRR	0	0	Inflammation
PRRs	0	0	Inflammation
RLR	0	0	Inflammation
RLRs	0	0	Inflammation
TH17	0	0	Inflammation
cdk	0	0	Proteomics
lacacystin	0	0	Proteomics
MMP2	0	0	Proteomics
NButGT	0	0	Proteomics
platelette rich plasma	0	0	Proteomics
caloric intake	0	0	Systemic
distance travelled	0	0	Systemic
exercising	0	0	Systemic
survivor	0	0	Systemic
enzymatics	0	0	Uncategorized
[Cu]	0	0	Chemistry
VGLUT	0	0	Excitability
BECN1	0	0	Apoptosis

Supplementary Table 2: The number of articles and figures per category.

Category	A	F
Axonal Transport	138	374
Chemistry	183	605
Energetics	496	1628
Excitability	295	942
Oxidative Stress	241	703
Inflammation	615	2138
Apoptosis	271	762
Proteomics	417	1463
Systemic	787	2283
